# From monolayer to organoids and multi-organ microphysiological systems: advancing regenerative medicine and precision therapies

**DOI:** 10.1186/s13287-026-05111-4

**Published:** 2026-07-02

**Authors:** Mahmood S. Choudhery, Ahmad Niaz, Taqdees Arif, Ruhma Mahmood

**Affiliations:** 1 Department of Human Genetics & Molecular Biology, University of Health Sciences, Lahore, Pakistan; 2 Allama Iqbal Medical College, Jinnah Hospital, Lahore, Pakistan

**Keywords:** Stem cell culture, Organoids, Organ-on-a-chip, Regenerative medicine, Precision medicine, Microphysiological systems

## Abstract

**Background:**

The landscape of in vitro models has evolved from simple two dimensional (2D) cultures to three-dimensional (3D) organoids and multi-organ microphysiological systems. Early monolayer cultures enabled directed differentiation but provided limited physiological relevance.

**Main Body:**

The development of organoid technology is a significant invention, which allows the cells to self-organize into complex 3D structures to recapitulate the cell diversity, architecture and functions of natural tissues. This has enabled more effective modelling of patient-specific diseases and processes. The latest development of the multi-organ microphysiological systems, integrates organoids or engineered tissues with microfluidic channels through which nutrients are perfused, and blood flow as well as mechanical stimuli are mimicked. This technology provides precise control over the tissue microenvironment to facilitate dynamic cell interactions and communication among different tissue types.

**Conclusion:**

These platforms more precisely mimic human biological processes, thereby improving disease modelling, drug screening, and the development of tissue grafts for regenerative therapies. This review discusses the evolution from 2D monolayer cell cultures, through the formation of organoids, to the engineering of organ-on-a-chip systems. It underlines how these technologies have advanced regenerative therapies by enhancing the ability to repair or replace damaged tissues and precision medicine through the creation of patient-specific disease models and personalized treatment strategies. Importantly, this review provides a comparative critical assessment of the functional capabilities, limitations, and translational readiness of each platform, identifying the specific contexts in which each system excels or falls short of its alternatives.

**Graphical Abstract:**

**Figure:**
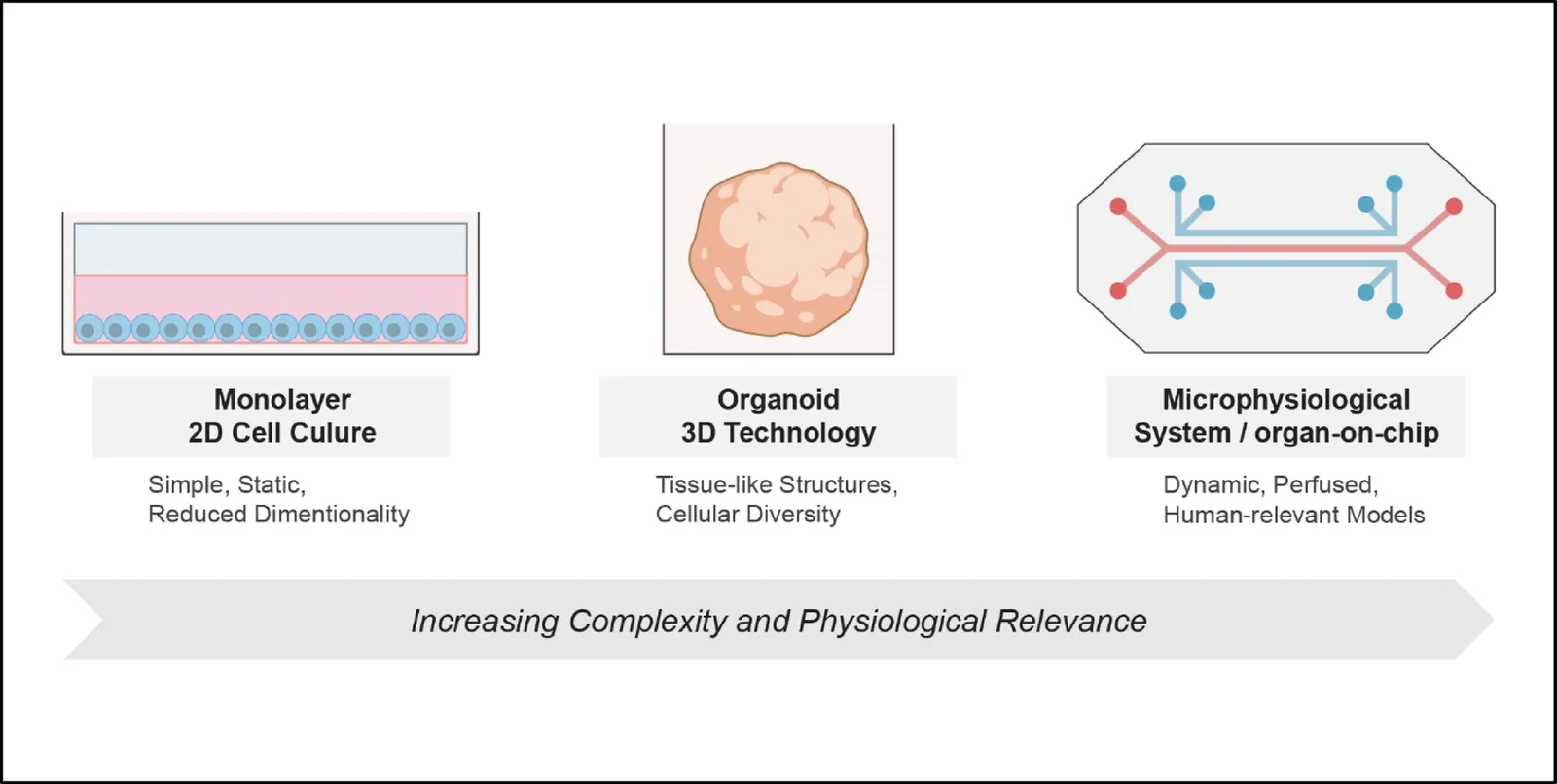

## Introduction

Cell culturing is the technique of growing cells under controlled in vitro conditions, offering a simple and potent approach for studying cellular functions and physiological mechanisms. Standard monolayer cultures are commonly used for basic cell biology research such as to study signaling and cellular interactions, to explore gene expressions and perform cancer studies. They also have applications in drug screening, toxicity assays, viral propagation, recombinant protein production and diagnostic development. However, their lack of spatial organization, tissue architecture, and dynamic microenvironmental signals, limit their ability to accurately mimic actual biological processes in vivo. In vitro, cell cultures are essential for scalable, reproducible, and ethically viable experimentation for simulating human development and medical conditions. Therefore, there was a critical need for more physiologically relevant models that can mimic the complexity of native tissues [1, 2], which has driven the emergence of stem-cell–based 3D culture systems.

Human embryonic stem cells (ESCs) and induced pluripotent stem cells (iPSCs) have become a source for both cell replacement therapy and modeling human development, enabled by breakthroughs in ESC isolation and reprogramming of somatic cells to pluripotent states [3]. Stem cell research has advanced our understanding of organogenesis by investigating pluripotent stem cell (PSC) self-organization in 3D culture. These advancements have resulted in the creation of organoids, which are cell culture systems capable of producing organ-like tissues [4].

Organoid technology has enabled stem cells to organize into three-dimensional(3D) structures that replicate organ-specific morphology, cellular diversity, and functionality. 3D organoids are self-organizing, multicellular constructs derived from PSCs or adult stem cells that mimic the structure and function of their in vivo counterparts more accurately than conventional monolayer cultures. This development of in vitro models from 2D monolayers to 3D organoids and microphysiological systems (MPSs) is an evolutionary change that has led to transformative advancements in regenerative medicine and precision therapies, by enabling patient-specific disease modelling, drug testing, and tissue engineering that are physiologically relevant.

The invention of patient-derived organoids (PDOs) has profoundly revolutionized cancer research and precision medicine, as these models improve the efficacy of conventional 2D monolayer cultures by precisely replicating the patient-specific genetic and phenotypic features. Their capability to maintain genetic integrity and enable prolonged proliferation in PDOs has enhanced their therapeutic significance for personalized treatment strategies.

Advancements in gene editing (CRISPR-Cas9), single-cell sequencing, and 3D bioprinting have markedly enhanced the functionality, scalability, and reproducibility of organoid cultures, thereby optimizing their utility for high-throughput drug screening and therapeutic validation [5]. Organoid technology has become essential for simulating human development, investigating genetic disorders, and evaluating therapeutic approaches tailored to individual patients. These models not only reduce the requirement of animal testing but also enhance translational accuracy for human biology [6]. Despite their significant potential in regenerative medicine and precision therapies, organoids have several limitations. These limitations include poor immune system, irregular vascularization and batch-to-batch variability. In addition, they may not fully recapitulate biomechanical signals or systemic interactions present in vivo. All these limitations make them difficult to scale and reproduce in a standardized, high-throughput way, which restricts their application in translational settings and pharmaceutical systems. MPSs also known as organ-on-chips (OoCs) were developed to overcome these limitations. These systems combine 3D bioengineered tissues or organoids with microfluidic devices in order to simulate human physiological functions [7]. These systems can mimic perfusion, mechanical cues, inter-organ communications and other parameters vital for tissue and organ physiology. Therefore, MPSs are better than conventional cell culture models. These systems may also be designed to create multiple organ-chip units that can be fluidically linked to create Body-on-a-Chip setups that recapitulate inter-organ interactions and whole-body physiology in miniature. In addition, they lower operational complexity and variability by supporting automated workflows, minimize human error, and significantly reduce reagent consumption due to the miniaturized volume requirements of microfluidic culture systems [8].

Several recent reviews have addressed individual aspects of this evolving landscape, including the integration of organoids with microfluidic platforms [7, 9], pharmacokinetic applications of MPSs with a focus on gut, liver, and kidney models [10], and the regulatory challenges associated with translating organ-on-chip data into drug approval frameworks [11]. However, these reviews predominantly focus on a single platform type or application domain, and a unified critical assessment of how each successive model addresses or fails to address the translational requirements of regenerative medicine remains lacking. This review traces the complete evolutionary trajectory from 2D monolayer cultures through 3D organoids to multi-organ microphysiological systems, evaluating the functional capabilities, limitations, and translational readiness of each platform. In addition, this review provides a comparative analysis that highlights the conditions under which each model outperforms or underperforms its alternatives, which is pertinent for guiding researchers and clinicians in selecting the most appropriate system for their experimental and therapeutic objectives.

## Monolayer (2D) cell culture

Two-dimensional (2D) cell culture has been a basis of biomedical research, for more than a century. This approach involves the proliferation of a single cell line on flat, adherent surfaces, such as petri dishes or culture flasks, maintained in a nutrient rich medium. This method is employed to examine the physiology of cells and tissues under medium that partially mimic in vivo environments. These investigations include studies of cell differentiation, migration, growth, physiological mechanisms, and cellular responses to biochemical alterations in their cultured environment [12]. The foundations of cell culture traced back to 1907, when Ross Harrison demonstrated that animal nerve cells could survive and extend axons outside the body, establishing the principle of in vitro growth. By the mid-20th century, the first immortal human cell line (HeLa, 1951) validated the long-term propagation of human cells. Since then, 2D culture has become integral to modern biology, pharmacology, and toxicology. Monolayer culture methods have been particularly important in stem cell biology field. The derivation of ESCs by Thomson et al. (1998) demonstrated that pluripotent cells could be maintained in monolayer conditions while retaining their ability to differentiate into all three germ layers. The subsequent development of iPSCs in 2006 further underscored the significance of 2D culture as a platform for generating patient-specific pluripotent cells, marking a pivotal advancement in regenerative medicine and personalized therapy [13, 14]. Thus, monolayer cell lines and primary cultures have provided foundational insights into cell biology under controlled laboratory settings, establishing a foundation for more complex tissue models.

### Advantages of 2D cell cultures

Traditional 2D culture is relatively simple and cost-effective supported by defined protocols for cellular proliferation and functional studies that are widely adopted across laboratories. Monolayers provide high reproducibility and facilitate easy manipulation such as gene transfection, pharmacological perturbation, and real-time imaging. Their compatibility with high-throughput formats makes them ideal for large-scale, high-throughput drug screening. Cells can be efficiently expanded on flat culture flasks, enabling easy microscopic observation of morphology, protein expression, and other phenotypes with minimal technical complexity and cost. Furthermore, since all cells in a monolayer are exposed to a homogenous media, the consistent availability of nutrients and growth factors promotes uniform growth and behavior throughout the culture. This simplicity, scalability to multi-well plate formats, and well-established protocols collectively illustrate why 2D culture remains a foundational in both academic and industrial research and development. These advantages have established 2D culture as the enduring backbone of in vitro biology [15, 16]. Notably, despite the emergence of more complex 3D platforms, 2D monolayer cultures continue to outperform better than organoids and MPSs in several critical contexts particularly in applications requiring target validation and analysis of signaling pathways, high-throughput drug screening, scalability for large datasets, and cost- and time-efficient assay development. For initial target validation and mechanistic analysis of individual signaling pathways, the controlled homogeneity of monolayers provides clearer cause-effect relationships that are often confounded by the heterogeneity inherent in 3D systems [15]. In high-throughput drug screening campaigns, 2D cell culture formats remain the practical standard for testing thousands of compounds quickly and cost-effectively. This is because current organoid and organ-on-chip platforms do not possess the necessary throughput capacity and automation compatibility for primary screens at this scale [17]. Furthermore, regulatory toxicology assays that rely on standardized, well-characterized cell lines are validated exclusively in 2D formats, and transitioning these to 3D models would require extensive re-validation [18]. Therefore, the selection of an appropriate culture system should be guided by the specific research question rather than a presumption that increased complexity necessarily translates to improved experimental outcomes.

### Limitations and translation challenges

**Fig. 1:**
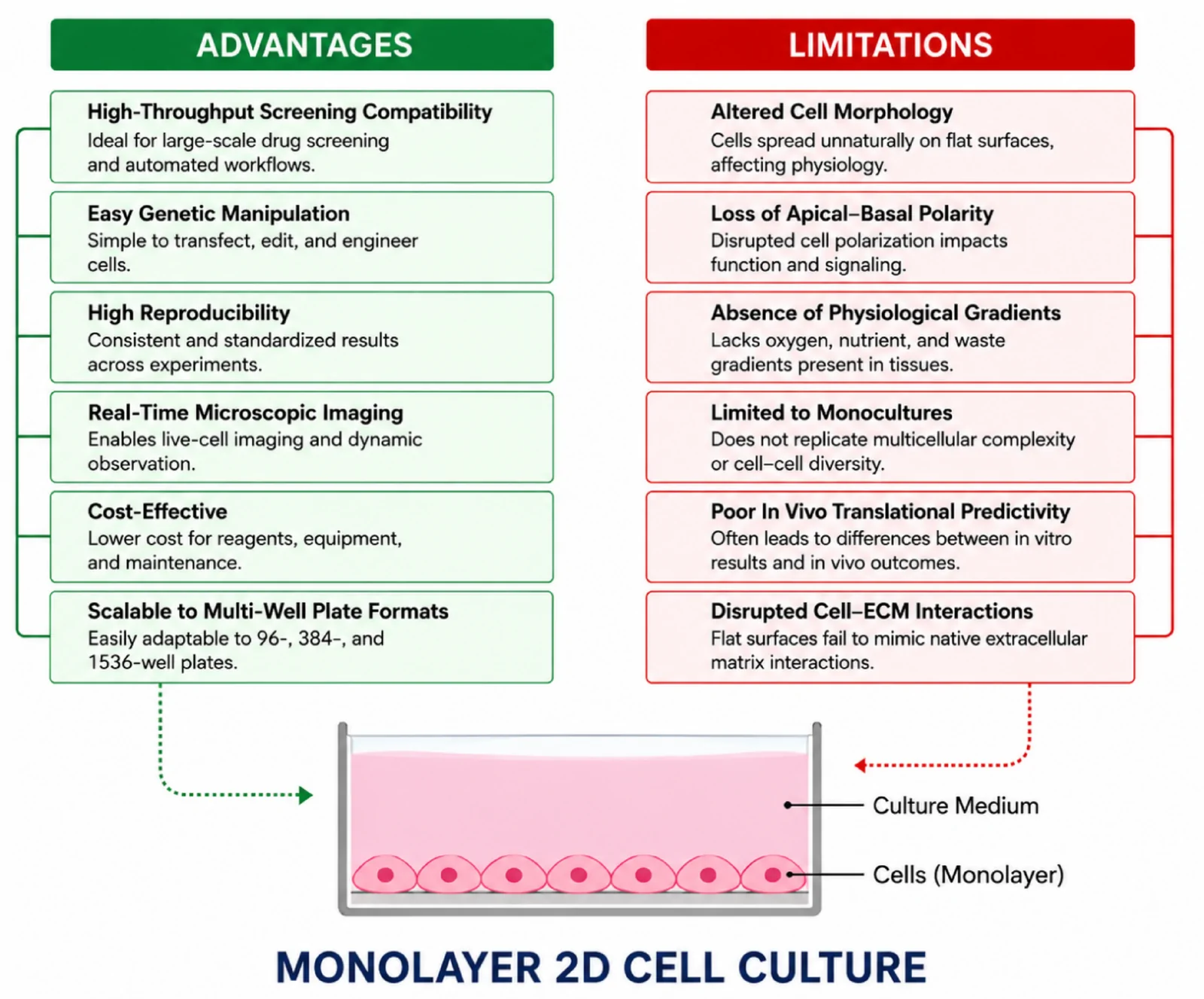
Advantages and limitations of 2D monolayer cultures. Conventional 2D systems support high-throughput screening, genetic manipulation, and reproducible experimentation. However, their simplified architecture results in altered cell morphology, disrupted polarity, absence of physiological gradients, and limited translational predictivity, contributing to poor correlation with in vivo drug responses

Despite their utility, monolayer cultures possess intrinsic limitations that restrict their physiological significance. A 2D culture, lacks the 3D architecture and complexity of real tissues, and therefore cannot fully reproduce the in vivo cellular microenvironment. They incompletely capture interactions among cells and between cells and the extracellular matrix (ECM). These interactions are critical for regulating cell differentiation, proliferation, viability, gene and protein expression, responsiveness to stimuli, drug metabolism, and various other cellular processes. Following isolation from the tissue and adaptation to 2D conditions, cells often undergo changes in morphology and division, frequently accompanied by a loss of tissue-specific phenotype. The altered morphology of the cells can influence their function, the organization of intracellular components, secretion, and cell signaling. Disruption of native ECM contacts causes adherent cells to lose polarity, which in turn modifies responses to cues such as pro-apoptotic signals. In addition, cells in a 2D monolayer are exposed to oxygen, nutrients, metabolites, and signaling molecules in a uniform manner, failing to reproduce the concentration gradients that exist in vivo [12, 19]. Moreover, adherent cultures are typically monocultures, limiting studies to a singular cell type (Fig. 1).

In addition to these biological limitations, monolayer cultures face translational challenges in transforming research outcomes into therapeutic applications. Conventional 2D methods have restricted spatial capacity and lack the dynamic gradients of nutrients, gases, and mechanical stimuli seen in vivo, which complicates long-term culture and scalability for clinical production. For example, muscle satellite cells (adult muscle stem cells) grown in 2D culture conditions rapidly exit quiescence and differentiate, thereby diminishing their engraftment capacity and ability to regenerate damaged muscle [20]. Similarly, multiple drugs that demonstrate efficacy in cancer cell lines in 2D cultures exhibit reduced potency in animal models or human patients because 2D cells fail to mimic the pharmacological response to 3D tumor tissue [1]. These variations highlight the limited predictive capability of simple monolayer studies for clinical outcomes. Consequently, there has been a rapid initiative to develop advanced culture systems that more accurately mimic human physiology. Advanced 3D platforms, such as organoids, and microfluidic organ-on-a-chip technologies increasingly address these limitations by providing more realistic tissue architecture and microenvironmental regulation [13].

## Organoid (3D) technology

The concept of “3D culture models” is often traced to assays developed by Barcellos-Hoff et al. (1989) and Petersen et al. (1992). Even earlier, floating collagen gels described in the 1970  s represented one of the first 3D culture systems. Before 2005, the term organoid generally referred to small, organized tissue fragments isolated from organs, primarily epithelial tissues, using mechanical and enzymatic digestion, and then grown in various 3D gels to form organ-like structures an advancement in 3D cultures [21]. Organoids maintain cell–cell and cell–matrix interactions within a matrix scaffold, typically ECM gels, resulting in the formation of several cell layers and internal lumens, which provide gradients of nutrients, oxygen, and signaling factors that are lacking in 2D culture. Consequently, organoids can differentiate into several cell types and structures analogous to those in vivo and more accurately mimic tissue-specific physiology and pathology compared to traditional flat monolayer cultures (Fig. 2).

### Methods of organoids generation

**Fig. 2:**
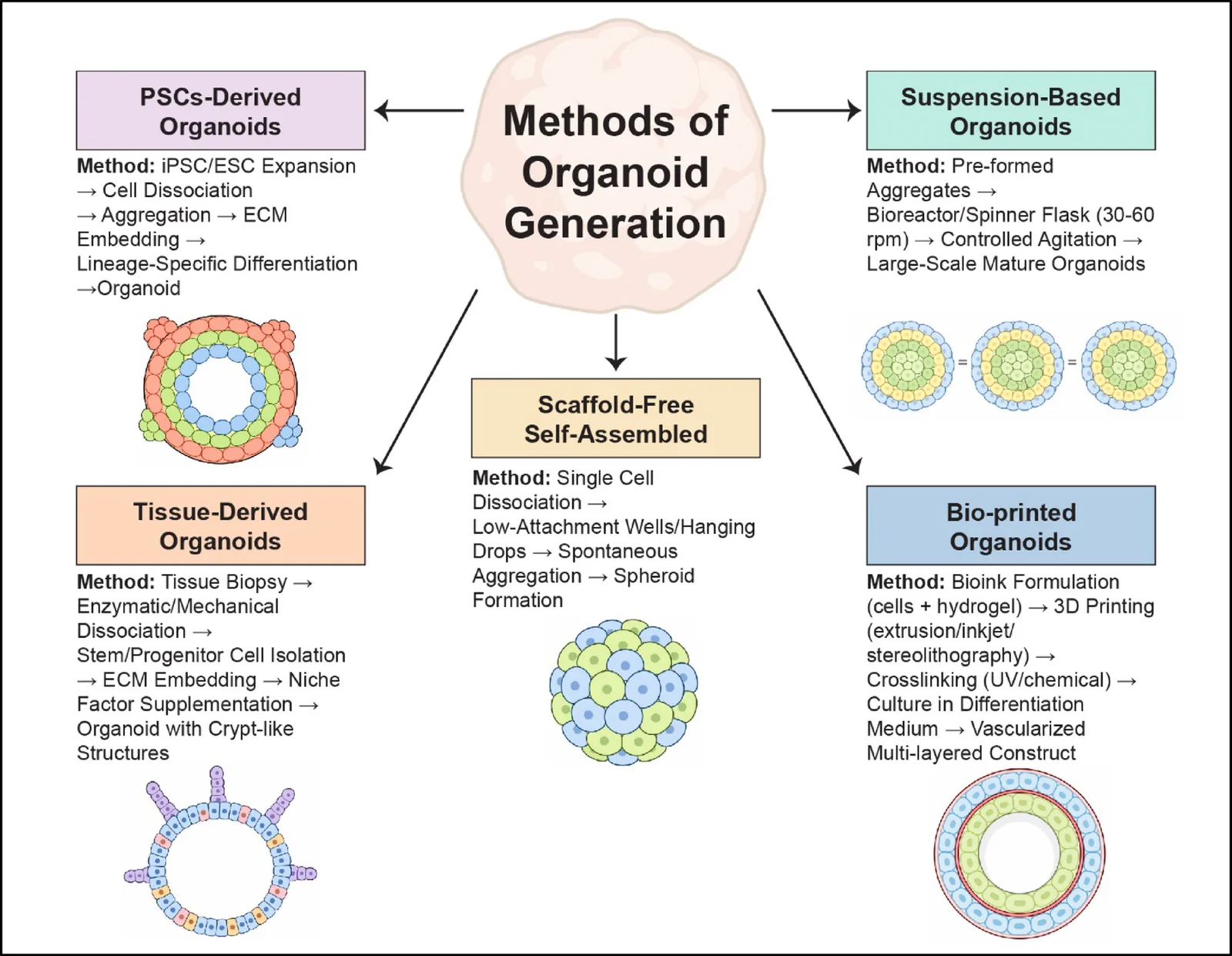
Methods of organoid generation. Organoids can be generated using pluripotent stem cells, tissue-derived adult stem cells, scaffold-free self-assembly, suspension-based dynamic culture, or bioprinting strategies. Each method differs in scalability, structural control, and maturation potential, providing complementary platforms for modeling development, disease, and regenerative processes

#### Pluripotent stem cells-derived organoids

In a standard PSC-derived organoid approach, iPSCs or ESCs are first expanded in 2D and then dissociated into single cells or tiny clusters for aggregation. To generate homogeneous embryoid bodies, cells are seeded into microwells, hanging drops, or low-attachment plates. After several days of aggregation, cell clusters are moved into a drop or bead of Matrigel or a comparable ECM. The Matrigel scaffold, rich in laminin, collagen IV, and growth factor–like proteins [22] facilitates cellular attachment and offers a 3D framework. Upon embedding, a lineage-specific differentiation medium is introduced, which comprises lineage-inductive signals pertinent to the specific organ type. For instance, cerebral organoid media incorporate dual-SMAD inhibitors and neurotrophic factors, whereas intestinal organoids utilize Wingless/integrated signaling pathway (Wnt) activators and epidermal growth factor (EGF) [23, 24]. The cells subsequently proliferate and self-organize within the matrix, creating complex tissue architecture. Over time, these structures acquire increasing cellular diversity and spatial patterning, although variability between batches remains a known limitation. To enhance nutrition and oxygen flow, long term organoid cultures frequently employ dynamic conditions such as orbital shakers or spinning bioreactors to prevent necrotic core formation and improve overall maturation.

#### Tissue-derived organoids

Organoids derived from adult stem cells follow a similar approach but start from primary tissue. Samples are enzymatically and mechanically dissociated to isolate stem/progenitor cells or small tissue fragments, which are then mixed with gel matrices, commonly Matrigel, in small domes or droplets. The culture medium is supplemented with critical niche signals: for several epithelial organoids, the media contains Wnt-pathway agonists (R-spondin1 or Wnt3a), EGF, and bone morphogenetic protein (BMP) inhibitors (Noggin or Gremlin 1). For example, mouse intestinal organoids proliferate vigorously in a media supplemented with R-spondin1, EGF, and Noggin. The media for human intestine and hepatic organoids often additionally require nicotinamide, TGF-β inhibitors (e.g., A83-01), or fibroblast growth factors (FGFs) to enhance longevity and promote maturation [24, 25]. Over the course of days to weeks, cultured cells proliferate clonally and undergo spontaneous differentiation. Intestinal organoids form luminal spheres with emerging crypt-like regions of proliferative stem cells, while some neural organoids develop ventricle-like cavities lined by neural progenitors [26]. Notably, differentiation trajectories and growth rates can vary considerably depending on donor age, tissue quality, and culture conditions. Organoids are maintained by partial medium changes and passaged via mechanical or enzymatic dissociation, depending on tissue type and desired yield, with long-term cultures periodically re-embedded to maintain structural integrity.

#### Scaffold-free self-assembled organoids

Scaffold-free organoids are 3D cellular aggregates generated without exogenous ECM materials, relying on cell–cell adhesion and endogenous ECM secretion for self-organization. This method involves the dissociation of PSCs, progenitor cells, or primary cells into individual cells, which are then permitted to reaggregate in non-adherent environments, like hanging-drop plates, round-bottom ultra-low attachment wells, or microwell arrays. The resultant aggregates spontaneously condense into spheroids in which cell polarity, differentiation, and tissue-like organization arise in response to intrinsic signaling cues rather than extrinsic scaffolding effects. The fundamental principle of scaffold-free spheroid culture relies on minimizing cell adherence to culture surfaces while enhancing the aggregation of suspended cells. This is accomplished via non-adhesive substrates, dynamic culture conditions, and the application of external stimuli such as rotational forces, magnetic levitation, or acoustic fields [27, 28]. Depending on the desired tissue lineage, culture media are supplemented with tissue-specific differentiation factors such as FGFs, Wnt activators, BMP inhibitors, or neurotrophic factors to promote lineage commitment and structural organization over time.

#### Suspension-based organoids

Suspension-based organoids are cultured and maintained in dynamic systems, such as spinner flasks, rotating wall vessels, or stirred-tank bioreactors. This method is especially efficient for large, metabolically active organoids, such as brain or hepatic models, that require improved nutrition and oxygen transport beyond the constraints of static culture. In a standard suspension technique, pre-formed aggregates or embryoid bodies derived from PSCs are transferred to bioreactors containing differentiation medium and cultivated under controlled agitation (usually 30–60 rpm). Gentle mixing reduces aggregate fusion, preserves spherical morphology, and reduces shear stress. The constant fluid movement enhances mass transfer, ensuring uniform nutrient distribution and the efficient elimination of metabolic waste. Media are routinely refilled or continually perfused via automated devices to sustain steady growth conditions. This approach facilitates larger-scale production, supports long-term maturation of organoids and reduces necrotic core formation, which is common in static ECM-embedded systems [29, 30]. However, agitation intensity, vessel geometry, and impeller design must be carefully optimized to prevent mechanical damage while maximizing mass transport.

#### Bio-printed organoids

3D bioprinting, originally developed as a method for fabricating solid scaffold by Charles Hull, has evolved into a powerful technology for spatially patterning biological materials, living cells, and growth factors. In 3D bioprinting, biomaterials and cells are precisely deposited in predefined architectures under computer control. It is a sequential integration of biomaterials and living cells within a defined 3D framework. This is achieved by depositing biocompatible hydrogels referred to as bioinks, into acellular and cellular frameworks using automated 3D printers. These bioinks typically consist of naturally derived polymers such as alginate, gelatin methacryloyl, or collagen, which support cell viability and tissue-specific differentiation. Recent advancements in 3D bioprinting for organoids or cellular aggregates have significant potential to address the challenge of producing large-scale tissue constructs for the fabrication of biomimetic complex tissues and organs in vitro [31]. In a typical workflow, high-density cell suspensions are combined with hydrogel-based bioinks and subsequently extruded or light-cured into specified architectures, such as tubular, multilayer, or lattice structures, using extrusion, inkjet, or stereolithography printers. After printing, the structures undergo crosslinking and are grown in lineage-specific differentiation conditions under static or dynamic perfusion culture to support maturation and promote vascularization (Table 1).

**Table 1:** Comparison of different methods of organoid generation

Organoid Type	Cell source & preparation	Key generation steps	Culture conditions & supplements	Structural support	References
PSCs-derived organoids	Human ESCs or iPSCs; dissociated at 70–90% confluence with ROCK (Rho-associated coiled-coil protein kinase)inhibitor	Aggregate formation → Stage-specific patterning → ECM embedding or suspension maturation	Dual-SMAD inhibition, Activin A, FGF, Wnt, BMPs; neurotrophins, antioxidants; shaking/bioreactor culture	Matrigel, PEG hydrogels, microwells	[23, 24, 26, 32]
Tissue-derived organoids	Biopsy or surgical tissue; mechanical and enzymatic dissociation to isolate stem/progenitor cells	Tissue isolation → ECM embedding → Niche factor supplementation → Passaging every 7–14 days	EGF, R-spondin1, Noggin; additives: B27, NAC, nicotinamide, Y-27,632 for single-cell survival	Matrigel domes, collagen; low-adhesion plates	[24, 25, 33]
Scaffold-free self-assembled organoids	PSC-derived or primary progenitors; prepared as single cells or clusters	Cell aggregation in suspension or microwells → Differentiation in lineage-specific medium	Lineage-specific media; B27/N2; controlled oxygenation; <500 μm diameter to avoid necrosis	None (self-assembly via adhesion)	[27, 28]
Suspension-based organoids	PSC aggregates or organoids preformed in microwells	Transfer to bioreactor → Continuous agitation → Differentiation and size monitoring	PSC-derived media; agitation-controlled O₂/pH; perfusion or periodic exchange	Spinner flasks, stirred-tank bioreactors	[29, 30]
Bio-printed organoids	PSCs, primary cells, or preformed organoids mixed with hydrogel bioinks	Bioink formulation → 3D printing → Crosslinking → Culture in lineage-specific media	GelMA, alginate, ECM gels; lineage media; printing optimized for pressure and viability	3D bioprinters, perfused or vascular designs	[31, 34]

### Methodological considerations for organoid generation

It is important to note that these five approaches including PSCs, tissue-derived adult stem cells, scaffold-free self-assembly, suspension-based dynamic culture, or bioprinting strategies of organoid generation are not separate categories; instead, they represent complementary methodological strategies that vary along two main axes: cell source (PSCs versus tissue-derived adult stem cells) and culture format (static ECM-embedded, scaffold-free, suspension-based, or bioprinted). Choosing the right method relies on the particular research context and the compromises associated with each option. For example, PSC-derived organoids provide extensive differentiation potential and are relevant for modelling early organogenesis, while tissue-derived organoids better maintain the adult stem cell niche and are thus preferred for patient-specific disease modelling, especially in oncology where preserving tumor heterogeneity is essential [33]. Scaffold-free approaches eliminate the perverse impact of exogenous ECM components, such as the batch variability and undefined composition inherent to Matrigel, which remains a persistent standardization challenge in the field [35]. Nonetheless, scaffold-free systems typically yield smaller and less architecturally intricate structures in comparison to ECM-embedded counterparts. Suspension-based culture in bioreactors overcomes the scalability and necrotic core issues of static methods, positioning it as the favored approach for large, metabolically active organoids like cerebral and hepatic models, though optimizing agitation parameters continues to be empirically challenging [29, 30]. Bioprinting provides unmatched spatial control over cellular positioning, which is particularly valuable for engineering vascularized or multi-layered constructs [31, 34], but it introduces additional complexity in terms of bioink rheology, cell viability during printing, and the specialized equipment required. Therefore, the selection of a generation method should be guided by the balance between structural complexity, reproducibility, scalability, and the specific biological question under investigation.

### Functional maturation and cellular diversity in organoids

The ability of organoids to produce many cell types from the target tissue is one of their distinguishing characteristics. For instance, a variety of neuronal subtypes are generated by PSC-derived neural organoids along with glial cells arranged into region-like domains, and adult intestinal organoids yield all of the epithelial lineages (absorptive cells, goblet, Paneth, and enteroendocrine) found in vivo. This cellular diversity results from intrinsic differentiation processes initiated during culture. However, organoid cells frequently exhibit characteristics similar to fetal-like or immature states rather than fully developed adult tissue. Transcriptomic analyses indicate that a brain organoid cultured for a few months resembles the mid-fetal human cortex, and it is only after prolonged culture (about 10 months) that the organoid aligns with late-prenatal expression profiles [24, 26]. Thus, organoids accurately replicate early development but achieve complete maturation only gradually under static culture conditions. Recent developments have demonstrated that bioengineering can improve organoid maturation. For example, engineered ECM scaffolds enriched with human fibronectin significantly expedited neural maturation: brain organoids generated on fibronectin matrices demonstrated enhanced neurogenesis, augmented glial differentiation, and sustained viability for over 7 months with improved structural complexity, including cerebrospinal fluid-like compartments [23].

Similarly, cardiac organoid maturation has emerged as a pivotal area of investigation, given the well-documented functional immaturity of iPSC-derived cardiomyocytes that limits their predictive value for drug screening and disease modelling. Ronaldson-Bouchard et al. demonstrated that initiating electromechanical stimulation at an early stage of iPSC-derived cardiomyocyte differentiation, with progressively increasing intensity over four weeks, yielded cardiac tissues exhibiting adult-like gene expression profiles, organized sarcomere ultrastructure with physiological sarcomere length (2.2 μm), the presence of transverse tubules, and positive force-frequency relationships [36]. Li et al. developed a closed-loop cardiac tissue system where spontaneous travelling wave pacing improved the sarcomeric and functional maturation of hiPSC-derived cardiomyocytes, enabling better evaluation of drug proarrhythmic potential consistent with clinical risk classifications [37]. Additionally, cardiac MPSs that integrate aligned iPSC-derived cardiomyocytes in perfused microfluidic chambers have shown pharmacological responses, including IC50/EC50 values for known cardiotoxic compounds, that align more closely with tissue-scale reference data than traditional 2D cellular assays [38]. Cardiovascular toxicity continues to be a major reason for drug withdrawals, representing around one-third of safety-related pharmaceutical withdrawals. This highlights the necessity of attaining adequate cardiac tissue maturation in organoid models to guarantee effective cardiotoxicity screening. Nonetheless, achieving complete adult cardiac maturation in vitro continues to be challenging, as most existing protocols result in fetal-to-neonatal phenotypes. The incorporation of various maturation cues, such as electrical stimulation, metabolic conditioning, and multicellular co-culture, may be necessary to reach adult-level functionality.

### Applications of organoids

#### Disease modelling

Organoids have transformed disease modelling by precisely mimicking human tissue architecture and functionality. Human cerebral organoids have been utilized to simulate neurotropic viral infections in studies of infectious diseases. The Zika virus infects cortical organoids, specifically killing neural progenitor cells and resulting in organoid atrophy, thus replicating the microcephaly-like cortical thinning observed in neonatal Zika syndrome [39]. Similarly, human lung and intestinal organoids have been used to model SARS-CoV-2 (COVID-19) pathology and test antivirals as they capture tissue-specific viral tropism and host responses, enabling high-throughput drug screening for COVID-19 treatments [40]. Organoids can be used in the study of genetic conditions; for example, patient-derived intestinal organoids from cystic fibrosis (CF) patients retain cystic fibrosis transmembrane conductance regulator (CFTR) mutation-specific functionality and have been employed to evaluate genotype-dependent treatment responses [41]. In each case, 3D organotypic cultures reproduce patient- and disease-specific phenotypes that are difficult to model in 2D cell cultures or animals. However, organoid-based disease models have significant limitations that restrict their translational utility. The lack of vascularization, immune cell populations, and stromal components indicates that organoids are unable to replicate the intricate multicellular interactions that influence disease progression in vivo, especially for conditions where the tumor microenvironment or immune response is crucial to the pathogenic process. The inherent variability of self-organization leads to significant inter-organoid diversity in size, morphology, and cellular composition, complicating the quantitative reproducibility needed for standardized disease phenotyping across laboratories.

#### Drug discovery and toxicology

Organoids are progressively employed for drug discovery and safety evaluation, providing human-relevant platforms for high-content phenotypic screening. For instance, de Poel et al. evaluated 1,400 Food and Drug Administration (FDA)-approved drugs in intestinal organoids generated from CF patients using a forskolin-induced swelling assay. This study revealed numerous modulators, including PDE4 inhibitors and established CFTR modulators, that restored CFTR function in rare CFTR genotypes [42]. Such studies show the utility of organoids for the repurposing and discovery of novel medicines in a patient-specific manner. Organoids also enhance toxicological predictions. A human liver organoid model incorporating patient-matched immune cells was used to investigate immune-mediated drug-induced liver injury. Co-culturing iPSC-derived liver organoids with autologous CD8^+^ T cells replicated the adaptive immune response to the antibiotic flucloxacillin in HLA-B*57:01 carriers. This method recapitulated T-cell proliferation, cytokine production, and hepatocyte apoptosis which reflects clinically observed immune-mediated toxicity [43]. Organoid-based assays can identify immune-mediated and tissue-specific toxicities that are often missed by traditional in vitro or animal models.

#### Precision medicine

Patient-derived organoids (PDOs) are potent tools for precision and personalized medicine, particularly in oncology. PDOs preserve the histological and genetic characteristics of the donor tumor, facilitating ex vivo drug testing tailored to the individual. Organoids generated from patients with malignant peritoneal mesothelioma maintained the characteristics of the original tumor, and drug-sensitivity studies conducted on these organoids accurately predicted each patient’s clinical treatment response [44]. In colorectal cancer, matched organoids derived from a patient’s primary and metastatic lesions were utilized in combinatorial drug screenings; despite their genomic similarities, the two organoids exhibited distinct sensitivity profiles to epigenetic drug combinations. This organoid-based approach identified individualized treatment vulnerabilities that would not have been apparent from DNA sequencing alone [45]. Despite these promising results, several translational barriers remain. The time required to set up and expand PDOs usually spans from two to eight weeks, depending on the type of tissue, which may exceed the clinical window for making treatment decisions in rapidly advancing cancers. Furthermore, while the alignment between organoid drug sensitivity profiles and actual patient clinical responses is promising in retrospective studies, it has not been confirmed through large-scale prospective clinical trials with adequate statistical power to establish PDOs as standard diagnostic tools in care.

#### Regenerative therapy

Organoid technology is progressing towards regenerative and transplantation applications. Recent research indicates that organoid grafts can successfully engraft and repair tissues in vivo. For example, human iPSC-derived kidney organoids infused into ex vivo perfused porcine kidneys and subsequently re-transplanted showed durable engraftment within renal tissue. Single-cell RNA sequencing, imaging, metabolic assays, and CRISPR–Cas9–engineered reporters revealed diverse transcriptional states and cell–cell interactions within the grafts [46]. Clinically, stem-cell-derived retinal organoid sheets have been transplanted into patients with retinitis pigmentosa. The grafts survived for more than two years, augmented retinal thickness at the transplantation sites, and mitigated vision deterioration, with no significant side effects reported [47]. These findings underscore the potential of organoids to restore function in damaged human tissues and to enable personalized regenerative therapies (Table 2).

**Table 2:** Comparative overview of in vitro culture systems

Features	2D Monolayer Culture	3D Organoids	Organ-on-Chip (OoC)
Cellular architecture	Cells flat on rigid plastic; single-cell monolayer, lacking native 3D structure.	Self-organize into multicellular 3D aggregates within an ECM scaffold; tissue-like architecture.	Engineered 3D microfluidic chambers with layered cell types
Physiological relevance	Low – no 3D cues or flow, so in vivo microenvironment poorly represented.	Moderate – ECM and 3D geometry partially restore physiological gradients and heterogeneity.	High – dynamic flow and mechanical cues recreate organ-level functions (e.g. breathing, shear forces).
Cellular interaction fidelity	Limited – mostly monocultures with forced polarity; lacks stromal or heterotypic contacts.	High – 3D geometry and ECM allow multiple cell types and native cell–cell/matrix interactions.	Very high – engineered co-cultures under flow precisely mimic tissue interfaces; enables complex cell–cell signaling.
Scalability/Throughput	Very high – amenable to 96/384-well automation and large-scale screens (standard 2D assays).	Moderate – spheroid formation can be high-throughput, but organoid protocols are laborious.	Limited – individual chips are low-throughput, though multiplexed microplate systems exist.
Dynamic microenvironment	None – static culture, no fluid flow or mechanical stimuli.	Minimal – typically static (nutrients/gases by diffusion only).	Yes – integrated perfusion and mechanical actuation (flow, stretch, and shear) mimic dynamic physiology.
Drug screening predictivity	Poor – often fails to predict in vivo responses (lack physiology leads to false positives).	Improved – organoids capture patient-specific responses	High – human chips reproduce clinical ADME (adsorption,distribution, metabolism and excretion)and pharmacodynamic responses, often outperforming animal models.
Technical complexity	Low – simple protocols (standard cell culture techniques).	Moderate – requires specialized matrices and culture conditions; complex differentiation protocols.	High – requires microfabrication, precise fluidic control and often biosensors.
Cost	Low – inexpensive plasticware and equipment.	Moderate – costly hydrogels, growth factors, and bioreagents.	High – device fabrication, pumps and sensors drive up cost
Limitations	Oversimplified environment, missing 3D cues and gradients.	Diffusion limitations (hypoxia/necrosis at core); lack vasculature and immune components.	Material issues (PDMS absorbs small molecules), complex setup, limited standardization.

## Microphysiological systems (MPSs)

A MPS/OoC is a microfluidic cell culture system designed using microchip manufacturing techniques, containing continuously perfused chambers seeded with living cells organized to mimic tissue and organ-level physiology. By generating multicellular architectures, tissue interfaces, physicochemical microenvironments, and vascular perfusion, these devices achieve tissue and organ functions not achievable with traditional 2D or 3D culture techniques. They provide high-resolution, real-time imaging and in vitro examination of biochemical, genetic, and metabolic activities of living cells within a functional tissue and organ framework. The first study demonstrating the OoCs concept was carried out by Huh et al., who introduced a lung-on-a-chip as a microfluidic in vitro system that simulates the physiology of a fundamental functional unit of an organ [48]. This method possesses significant promise to enhance the investigation of tissue formation, organ function, and disease origins.

### Principle of microfluidic technology

Microfluidic OoC devices manipulate very small volumes of fluid, typically from microliters to femtoliters, within channels ranging from tens to hundreds of micrometers in dimension. Microchannels of this dimension provide a high surface-area-to-volume ratio that enhances mass transfer, enabling rapid mixing and efficient exchange of solutes. Additional intrinsic advantages of microfluidics include accelerated mixing rates, enhanced volume control, and characteristics such as minimal reagent consumption and rapid response times [49, 50]. This facilitates precise regulation of concentration profiles; for instance, secreted factors can be maintained in diffusion-dominated flow or removed by convective flow through modulation of flow rate and channel geometry. Standard driving forces including capillary action, syringe pumps, basic gravity-fed or rocking systems, can ensure stable perfusion. Microfluidics has significantly influenced biological research by integrating into sample preparation methods and many cell culture applications.

#### Design and flow characteristics

The design of microchannels influences fluidic behavior. Networks of parallel and branching channels can replicate vascular beds and establish specific shear stress patterns. By altering channel dimensions and flow rates, OoC devices can create physiologically suitable shear conditions (for blood vessels) or low-flow scenarios (interstitial flow) for resident cells. The elevated surface-area-to-volume ratio in microfluidic devices facilitates efficient mixing, enabling the quick establishment of chemical gradients or oxygenation within tissue constructs. These features significantly decrease reagent and cell consumption, facilitating high-throughput investigations with minimal quantities.

#### Materials and fabrication methods

The majority of OoC devices are constructed using microfabrication methods such as photolithography, injection molding, hot embossing, and 3D printing. A lithographically printed mold, commonly composed of silicon or SU-8, defines channel shape; an elastomer, usually polydimethylsiloxane (PDMS) is embossed from this mold [51]. Increasingly, 3D printing technologies such as stereolithography and digital light processing are being utilized to fabricate these master molds directly, bypassing the need for cleanroom-based photolithography and substantially reducing prototyping time and cost while maintaining sufficient channel resolution for most OoC geometries [52, 53]. PDMS is widely used for its transparency, flexibility, gas permeability and biocompatibility. However, it has drawbacks including absorption of tiny hydrophobic molecules of drugs due to its hydrophobic nature, thereby distorting assay results. To resolve this issue, chips may undergo surface treatments (plasma, ECM coatings, lipophilic polymers) or be fabricated from alternative materials such as cyclic olefin copolymer (COC) or glass. Quintard et al. constructed their vascularized tissue chip using COC to enhance optical clarity and reduce absorbance [54]. Modern platforms can even integrate in situ sensors (electrochemical, electrical or optical) into the chip walls during fabrication, enabling real-time monitoring of pH, metabolites and barrier function without disrupting the tissue.

### Biomechanical cues and fluid dynamics

Microfluidic chips can replicate the mechanical microenvironment of tissues. Cells are responsive to fluid shear stress, mechanical strain, and pressure, and OoC systems can deliver these stimuli in a controlled way. A specified shear stress is applied to cells, such as endothelium, by circulating culture media through microchannels. The intensity of shear can be adjusted through channel geometry and flow rate. For instance, multiplexed channel configurations enable the simultaneous testing of several shear intensities [55]. Fluid dynamics inside OoC systems may simulate blood circulation, interstitial flow, or other physiological fluid movements. For instance, controlled continuous flow will expose endothelial or epithelial barriers to certain shear stresses (ranging from 0.1 to 10 dyn/cm² for microvasculature). This can be modified within a single device by engineering channels of varying widths or by employing flow dividers. In practice, the application of shear stress, even at small levels, frequently results in improved cell differentiation and barrier maturation. In the human heart-on-chip, endothelial cells subjected to medium flow exhibited alignment and established tight CD31-positive connections, but static cultures lacked this organized morphology [56].

In addition to fluid shear, numerous OoC systems apply dynamic mechanical strain to replicate tissue motions. A typical example is the lung-on-chip, where flexible PDMS membranes are cyclically elongated (via vacuum or pneumatic actuation) to simulate respiratory movements. A human alveolus-on-chip contained an air-liquid interface with alveolar epithelial cells on one side of a porous, flexible membrane and capillary endothelial cells on the opposite side. This device was used to replicate the approximately 10–20% cyclic strain of alveolar expansion during respiration [57]. Similarly, gut-on-chip devices often include lateral vacuum chambers or elastic walls to create peristaltic-like forces, and cardiac chips may use pacing or cyclic pressure to simulate heartbeats. In each case, these cyclic mechanical cues enhance tissue phenotypes.

### Microphysiological systems vs. organoids

While organoids and MPSs are often viewed as complementary platforms, an in-depth comparison shows that each system has distinct advantages and limitations that influence its suitability for particular applications. Organoids are highly effective in mimicking tissue-level self-organization and developmental processes, as they depend on inherent differentiation programs that create cellular diversity and spatial arrangement without the need for external manipulations. This quality renders them especially useful for modeling organogenesis, genetic disorders, and patient-specific tumor biology [4, 7]. However, organoids lack the controlled fluid flow, defined mechanical stimuli, and inter-organ communication that are essential for accurately modelling systemic pharmacokinetics, vascular transport, and biomechanical responses. MPSs address these limitations by providing precisely engineered microenvironments with continuous perfusion, controlled shear stress, and mechanical actuation, which are critical for applications such as drug metabolism studies, barrier function assessment, and cardiotoxicity screening [18, 48]. Conversely, OoC systems typically rely on externally patterned cell populations rather than self-organized architectures, which limits their capacity to model developmental complexity and often requires predefined cellular compositions that may not capture the full heterogeneity of native tissues.

The integration of organoids into microfluidic OoC systems has emerged as a rapidly advancing strategy that combines the biological fidelity of organoid self-organization with the environmental control of microfluidic engineering, recognizing the complementary strengths of both platforms [9]. In such organoid-on-chip configurations, pre-formed organoids are embedded within microfluidic chambers and subjected to continuous perfusion, which enhances nutrient delivery, reduces necrotic core formation, and enables real-time sampling of secreted biomarkers. For instance, vascularized organoid-on-chip platforms have demonstrated functional anastomosis between organoid-derived endothelium and engineered microvasculature, enabling intravascular perfusion and improved long-term culture viability [54]. Nevertheless, significant technical challenges remain in this integration, including the difficulty of standardizing organoid loading and positioning within microfluidic chambers, the variability in organoid size that complicates uniform perfusion, and the limited compatibility of current OoC materials with the long-term culture conditions required for organoid maturation (Fig. 3).

### Examples of microphysiological systems

OoC technology has been applied to virtually every major human organ. Here are the representative examples in the tabular form (Table 3).

**Table 3:** Overview of different organ-on-chip systems

Type	Cell source	Microfluidic features	Physiological mimicked features	Primary applications	Advantages	Limitations	References
Lung-on-a-Chip	Human primary alveolar epithelial cells + lung microvascular endothelial cells	Thin, stretchable membrane with cyclic strain (breathing motion); air–liquid interface and fluid shear	Alveolar–capillary gas-exchange barrier under physiological stretch	Pulmonary disease modeling, inhalation toxicity, drug testing	Recapitulates breathing movements and barrier function	Limited immune cell repertoire; PDMS absorbs small hydrophobic molecules	[48, 57–59]
Liver-on-a-Chip	Human hepatocytes (primary or iPSC-derived) co‑cultured with non-parenchymal cells	Perfused microchannels mimicking sinusoidal flow; includes zonation gradients	Hepatic lobule architecture with portal-central flow and oxygen gradient	Drug metabolism and hepatotoxicity screening, non-alcoholic fatty liver disease/fibrosis models	Maintains hepatocyte functionality and zonal metabolism	Complex co-culture setup; limited long-term viability; PDMS drug absorption affects metabolic assays	[60–63]
Heart-on-a-Chip	Human iPSC-derived cardiomyocytes, cardiac fibroblasts, and endothelial cells	Microfluidic chambers with flow, elastic posts, mechanical and electrical stimulation	3D aligned cardiac muscle with vascular endothelial layer under flow	Cardiotoxicity testing, cardiac disease modeling, electrophysiology	Multicellular structure and maturation; flow supports alignment	Incomplete maturation; lacks full conduction system	[36, 38, 56]
Brain-on-a-Chip	Human brain microvascular endothelial cells, pericytes, and astrocytes	Parallel channels with semi-permeable membrane; continuous perfusion; ECM gel in brain side	Blood–brain barrier tight junctions, polarized astrocyte end-feet	CNS drug permeability, neuroinflammation and neurodegeneration studies, nanoparticle transport	Tight junction fidelity and polarization	No full neural network; PDMS adsorption; limited long-term stability	[64–67]
Kidney-on-a-Chip	Human proximal tubule epithelial cells and glomerular elements (podocytes, etc.)	Compartmentalized channels with porous membrane and shear flow	Proximal tubule reabsorption and glomerular filtration mimicry	Nephrotoxicity assays, kidney disease models	Shear-enhanced function; mimics filtration	Simplified nephron; complex device alignment	[68–71]
Gut-on-a-Chip	Human intestinal epithelial cells ± stromal/immune cells and microbiome	Dual-channel design with porous membrane separating epithelial and vascular compartments; peristaltic-like stretch and flow	Intestinal villus structure, mucus layer, microbiome interactions	inflammatory bowel disease (IBD)/infection modeling, oral drug absorption, microbiome–drug studies	Captures peristalsis and microbiome interaction	Difficult microbiome maintenance; PDMS absorption; immune cell limitations	[72–75]
Tumor-on-a-Chip	Patient-derived tumor cells with stromal/immune cells	3D ECM microchambers ± vascular flow channels for invasion/metastasis	Tumor microenvironment: heterogeneity, hypoxia, perfusion gradients	Drug screening, immunotherapy models, metastasis and invasion tracking	Patient heterogeneity, dynamic exposure, real-time imaging	Capturing immune infiltration and metastasis is challenging	[76–78]
Body-on-a-Chip	Multiple organ-specific cultures (gut, liver, kidney, etc.)	Interconnected fluidic modules with recirculating media	Whole-body pharmacokinetics; organ–organ axis physiology	Systemic drug testing, multi-organ pharmacokinetics, gut-liver axis models, drug-drug interaction studies	Inter-organ crosstalk; human pharmacokinetics	Complex integration and scaling	[18, 61, 79]

## Applications of microphysiological systems

**Fig. 3:**
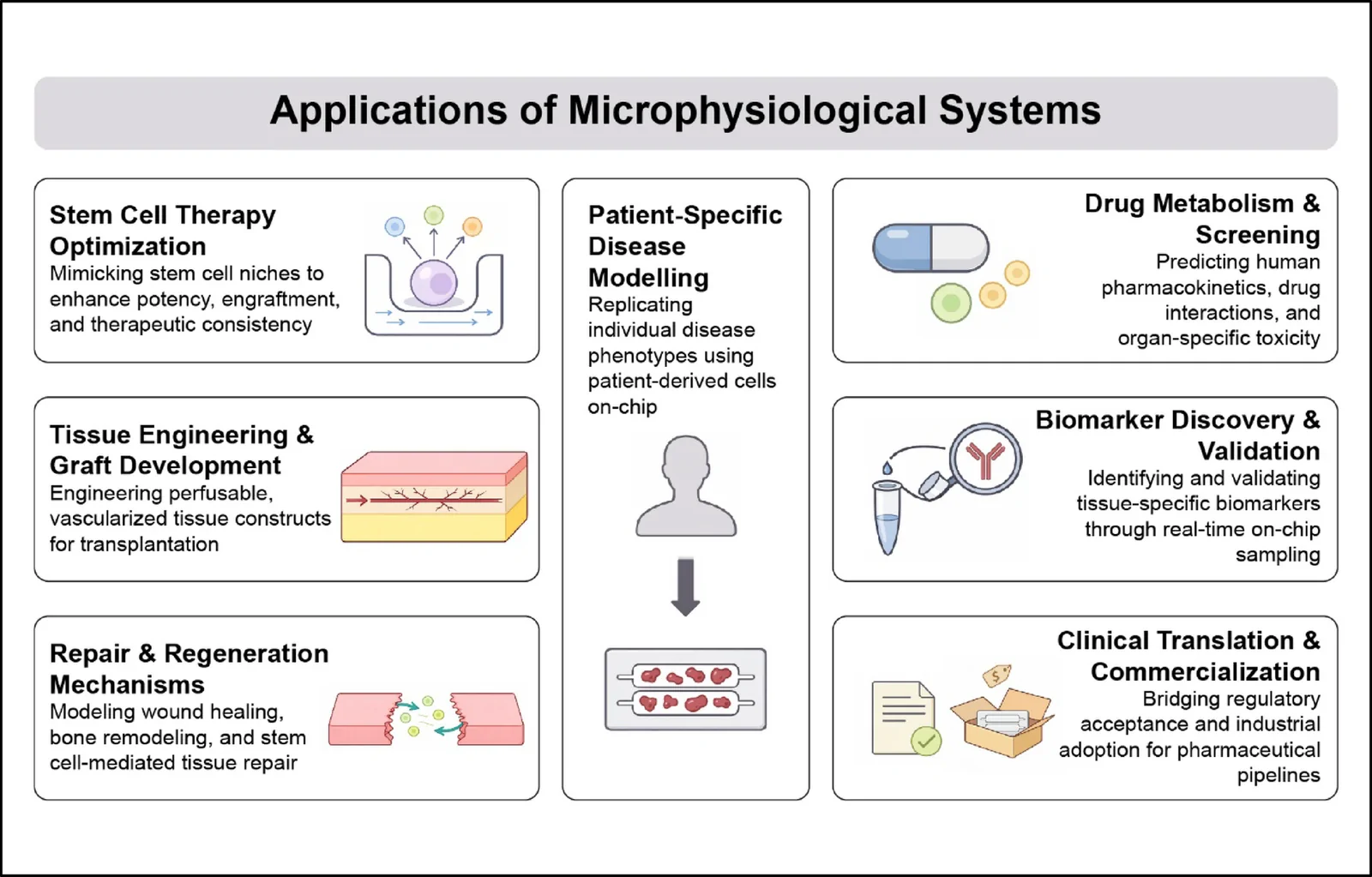
Major applications of microphysiological systems. Organ-on-chip platforms are applied across stem cell therapy optimization, tissue engineering, regenerative medicine, disease modeling, pharmacokinetic screening, and biomarker discovery. Their ability to integrate perfusion, multicellular complexity, and patient-specific cells enables predictive, human-relevant modeling for translational research

### Stem cell therapy optimization

OoCs provide more precisely adjustable microenvironments that accurately mimic stem cell niches, providing effective platforms for improving the quality, potency, and consistency of stem cell-based therapies. Sharipol et al. developed a bone-marrow-microenvironment-on-chip that included osteoblastic, endothelial, and mesenchymal stromal layers, which sustained hematopoietic stem and progenitor cells for more than 14 days in vitro. The platform retained roughly 0.3% Lin⁻Sca-1⁺c-Kit⁺ (LSK) cells, nearly doubling the in vivo frequency by approximately 0.15%. Moreover, competitive transplantation experiments validated that chip-derived CD45.2⁺ hematopoietic stem cells demonstrated strong multilineage engraftment comparable to natural bone marrow hematopoietic stem cells. This research presents a physiologically appropriate quantitative approach for assessing hematopoietic stem and progenitor cell-niche interactions and hematopoietic functioning in vitro [80]. Ajalik et al. reviewed MPS platforms designed to simulate musculoskeletal tissues and joint disorders. Only ~10% integrated immune or mechanical-loading modules, and <5% operated at high throughput (>96 devices). Most systems consisted of 2–4 patterned cell types within 20–50 mm² chips, with only approximately 12% attaining quantitative, multichannel readouts. In a 3D perfused skeletal muscle chip, human mesenchymal stem cell-derived myogenic precursors and iPSC-derived motor neurons were co-cultured under dynamic flow and exhibited enhanced alignment and maturation, with bigger myotubes, greater contractile calcium transients, relative to static cultures [81]. Nguyen et al. created a multi-organ-on-a-chip model utilizing human kidney and liver organoids, which preserved the physiological functions of both organoids and replicated renal damage through H₂O₂ exposure. Small extracellular vesicles produced from mesenchymal stromal cells, with a modal size of around 149 nm and a density of 1.13–1.14 g/ml, promoted epithelial regeneration in injured kidney tubuloids and exhibited enhanced accumulation in both wounded kidney and co-cultured liver organoids. This human-derived multi-organ-on-a-chip replicates the therapeutic efficacy and biodistribution of small extracellular vesicles observed in animal models, providing a semi-systemic in vitro platform for evaluating extracellular vesicle-based therapies [70]. However, the translation of these OoC-optimized stem cell products into clinical therapies remains constrained by the difficulty of scaling chip-derived cell populations to therapeutically relevant quantities, the limited standardization of chip-based potency assays, and the absence of regulatory frameworks specifically designed to qualify OoC-conditioned cell products for clinical use.

### Tissue engineering and graft development

The integration of tissue engineering and OoC technologies has facilitated the development of perfusable, multilayered, and physiologically mature structures, enhancing the production of transplantable tissue grafts with improved integration and functionality. Initial skin-on-chip models integrated an endothelium to replicate perfused vasculature. For instance, Wufuer et al. developed a three-layer human skin chip featuring microfluidic channels that facilitated endothelium and fibroblast networks beneath keratinocytes. This prototype, despite limited perfusion, illustrated the viability of integrating 3D skin structures with microvascular design [82]. Subsequent platforms showed true vessel integration. For example, Mori et al. engineered a full-thickness skin equivalent with perfusable endothelial-lined vascular channels within a microfluidic device, maintaining dermal and epidermal morphology, establishing endothelial tight junctions, and supporting nutrient delivery and molecular penetration from epidermis into vascular channels [83]. Similarly, Salameh et al. engineered a perfusable vascularized full-thickness human skin model with three macro-vessel channels connected to a microvascular network. Under perfusion, the construct showed physiological epidermal differentiation and barrier properties, providing a quantitative platform for topical and systemic testing [84]. Zimoch et al. developed a tri-layered human skin substitute (TLSS) consisting of an epidermis, pre-vascularized dermis, and an adipocyte-rich hypodermis sourced from human adipose stem cells. Adipose-derived stem cells differentiated into mature adipocytes that expressed adiponectin, FABP-4, and perilipin within a collagen I hydrogel, while the pre-vascularized dermal layer facilitated a dense capillary network. Upon transplantation into immunocompromised rats, the TLSS demonstrated a functional stratified epidermis, preserved hypodermal architecture, and regulated keratinocyte maturation through increased TGF-β1 release from hypodermal cells [85]. Therefore, OoC-derived grafts with built‐in vasculature can overcome diffusion limits and improve engraftment.

### Repair and regeneration mechanisms

Microfluidic platforms provide dynamic and bio-simulated environments that enable researchers to analyze the spatiotemporal processes involved in tissue repair and regeneration, providing unparalleled understanding of wound healing, organ remodeling, and stem cell-mediated regeneration. Sharma and Kishen developed a four-channel microfluidic “wound-on-a-chip” model for diabetic chronic wounds, incorporating human dermal fibroblasts and macrophages within a collagen I matrix, alongside endothelial cells in Matrigel, under hyperglycemic conditions combined with advanced glycation end products and lipopolysaccharide. This system replicated key characteristics of non-healing diabetic ulcers, including compromised ECM remodeling, diminished angiogenesis, increased levels of IL-1β/TNF-α/MMP9, and the induction of endothelial-to-mesenchymal transition [86]. In a kidney-tubule chip, transient oxidative injury (H₂O₂ exposure) was applied to human renal tubuloids. Subsequent perfusion with mesenchymal stromal cell-derived extracellular vesicles rapidly recruited vesicles to damaged epithelium and restored barrier integrity and solute transport to baseline [70]. Kim et al. developed a bone-on-a-chip platform that incorporates triply periodic minimal surface 3D scaffolds with controlled interstitial perfusion to investigate the effects of pore geometry and wall shear stress on osteogenesis. They demonstrated that scaffolds with intermediate solidity optimized wall shear stress distribution, resulting in significantly higher alkaline phosphatase activity and calcium deposition. A crucial pore size barrier was identified, beyond which flow-mediated signaling diminishes. This geometry-guided approach provides a quantitative mechanobiological framework for scaffold design in bone tissue engineering [87].

### Patient-specific disease modelling

MPSs are enhancing personalized treatment by incorporating patient-derived cells into microengineered tissues, thereby accurately replicating unique disease phenotypes and response patterns in vitro across various diseases. Özkan et al. developed human colon-derived organ-chip models integrating epithelial and stromal/fibroblast compartments sourced from either healthy donors or individuals with IBD. IBD-derived fibroblasts induce barrier dysfunction, increased cytokine (IL-6, IL-8, and TNF-α) release, and fibrotic ECM remodeling. Chronic carcinogen exposure caused genome-wide CNAs (copy number alterations) and TP53 mutations selectively in IBD chips. The study mechanistically links stromal inflammation, mechanical stress, and hormonal cues to IBD-associated tumorigenesis in a quantitative microphysiological model [73]. Plebani et al. engineered a microfluidic airway-on-a-chip using primary human ΔF508 CFTR-mutant bronchial epithelial cells at an air-liquid interface, complemented by perfused pulmonary microvascular endothelium. The CF model recapitulated typical signs of disease including thicker mucus layer, increased ciliary density and higher ciliary beating frequency compared to control chips. CF chips exhibited markedly increased IL-8 concentrations, which corresponded with enhanced neutrophil adherence, transmigration across the endothelium–epithelium interface, and increase in bacterial (Pseudomonas aeruginosa) growth rate relative to healthy airway chips. This human lung microphysiological device offers a quantitative, patient-relevant instrument for investigating CF pathogenesis and treatment evaluation [59]. Lall et al. developed a microfluidic “spinal-cord-chip” that incorporates iPSC-derived spinal motor neurons from sporadic Amyotrophic Lateral Sclerosis patients and features a vascular channel lined with brain-microvascular endothelial-like cells. The platform improved neuronal development, demonstrating gene expression variations and a particular dysregulation of glutamatergic and synaptic signaling. Neurofilament levels increased in patient samples, and motor neuron subpopulations exhibited a disrupted excitatory/inhibitory balance. This organ-chip mimics early sporadic Amyotrophic Lateral Sclerosis molecular characteristics and offers a functioning human model for motor neuron diseases [66]. Organ chips have enabled modeling of rare genetic conditions by using patient iPSCs. Nevertheless, patient-specific OoC models face practical challenges including the lengthy iPSC derivation and differentiation timelines, the high cost per patient model, and the current lack of standardized benchmarking criteria to validate whether chip-based disease phenotypes faithfully predict individual clinical trajectories.

### Drug metabolism and screening studies

Organ-on-chip technologies provide dynamic drug testing and real-time assessment of pharmacokinetics, pharmacodynamics, and metabolism, providing a more predictive and human-relevant alternative to conventional static cultures or animal models. In order to reproduce therapeutically relevant drug-drug interactions (DDIs), Lee-Montiel et al. developed isogenic human iPSC-derived liver and cardiac MPSs from the same genetic background. In this two-organ system, ketoconazole inhibited the liver module’s CYP3A4-mediated cisapride metabolism, which resulted in higher cisapride levels and a notable prolongation of the heart module’s cardiac action-potential duration (cAPD₉₀). The study found a margin of safety in comparison to human therapeutic concentrations and measured an EC₅₀ of roughly 9.6 nM for cisapride-induced cAPD₉₀ prolongation. This integrated hiPSC-based platform showed promise as a quantitative in-vitro model for human drug safety evaluation by correctly predicting the pharmacokinetic–pharmacodynamic interplay that underlies cisapride-associated cardiotoxicity [61]. Abbas et al. developed a primary human gut–liver microphysiological system to model first-pass metabolism and absorption. Perfusion of intestine and liver tissues with midazolam and other drugs yielded estimates of fraction absorbed, first-pass extraction, and oral bioavailability closely matching clinical data [62]. These models facilitate mechanistic in vitro predictions of drug bioavailability and metabolite profiles. Other organ chips have focused on specific barriers and metabolic processes, for example, blood-brain barrier chip model was utilized to assess the permeability of ten benchmark substances, with the observed permeability trends on-chip demonstrating a strong correlation with in vivo and animal data [65]. Similarly, Wang et al. developed an 18-organ microphysiological system with integrated vascular and excretion routes. This technique preserved tissue viability for almost two months and replicated compartmental pharmacokinetics. Significantly, it facilitated simultaneous monitoring of drug distribution in relation to organ-specific toxicity (e.g., metabolic, cardiac, hepatic, etc.) and could simulate complex scenarios such as multimorbidity and polypharmacy [79].

Despite these advances, a critical limitation of current OoC-based drug screening is the low throughput relative to conventional high-throughput screening platforms. Standard pharmaceutical HTS campaigns routinely test compound libraries of thousands to millions of molecules using automated 96-, 384-, or 1536-well plate assays, whereas most OoC systems operate at scales of fewer than 10 to 20 parallel chips per experiment, with manual handling steps that impede scalability [88]. The integration of robotic liquid handling systems, automated perfusion controllers, and high-content imaging platforms is increasingly recognized as essential for bridging this throughput gap. Several commercial OoC platforms have been developed to address this limitation: the MIMETAS OrganoPlate employs a membrane-free, gravity-driven 96-well microfluidic format compatible with standard plate readers and automated liquid handlers [89]. Emulate’s organ-chip platform has been validated in collaboration with the FDA for liver and intestinal toxicity screening and recently launched a 96-sample high-throughput system [90]. Nevertheless, even these advanced platforms remain orders of magnitude below the throughput of conventional well-plate HTS, which indicates that OoC systems are currently best suited for secondary and tertiary screening of pre-selected compound libraries, detailed mechanistic follow-up studies, and patient-specific validation, rather than for primary large-scale compound library screening of extensive compound libraries.

### Biomarker discovery and validation

Organ-on-chip systems allow controlled replication of physiological conditions and continuous sampling, making them ideal for identifying and validating biomarkers linked to tissue-specific responses. For example, tumor microenvironment-on-chip models have uncovered novel biomarker targets. Shen et al. developed a tri-cell co-culture microfluidic “tumor-on-a-chip” model for Hepatocellular carcinoma, integrating cancer cells, stromal hepatic stellate cells (HSCs), and endothelial cells. Transcriptomic and cytokine array analyses identified the iron-binding protein Lipocalin-2 (LCN2) as a crucial mediator of tumor microenvironment remodeling, targeted suppression of LCN2 reduced angiogenesis, restored sorafenib sensitivity, and enhanced NK-cell cytotoxicity. This microphysiological platform offers a quantitative approach to analyze stromal effects on tumor growth and treatment response [77]. In renal MPS models, conventional injury biomarkers can be quantified with improved sensitivity. A 3D proximal tubule chip generated from human kidney organoids exhibited a significantly elevated release of lactate dehydrogenase (LDH, a marker of cytotoxicity) upon exposure to nephrotoxic agents (cisplatin and aristolochic acid) relative to control chips, which correlated with its increased expression of drug transporters [69]. This demonstrates that perfused kidney chips can both detect known kidney-injury biomarkers and reveal physiologically relevant transporter-mediated toxicity. Likewise, Koenig et al. developed a human bone-marrow microphysiological system that includes a perfused ceramic scaffold seeded with mesenchymal stromal cells and CD34⁺ hematopoietic progenitors, promoting erythroid, myeloid, and natural killer cell development for 31 days. The system maintained multilineage output and responded to pro-inflammatory cytokines. Furthermore, in an autologous setting utilizing peripheral-blood T cells, administration of T-cell bispecific antibodies prompted T-cell activation and target-cell destruction, indicative of on-target off-tumor toxicity [91]. This platform provides a quantitative platform for hematologic safety assessment of biologics.

### Clinical translation and commercialization

As regulatory agencies and pharmaceutical companies increasingly validate OoC systems, these technologies are closing the translational gap by enhancing predictive accuracy for human responses, improving drug safety evaluations, and guiding clinical trial design. The OoC and organoid technologies are merging to create advanced MPSs that more accurately replicate human tissue architecture, dynamic mechanical stimuli, and inter-organ interactions compared to conventional static or animal models. Over 90% of drug candidates fail in clinical trials due to insufficient preclinical models, but OoC platforms now provide the quantifiable modeling of complicated diseases, host-microbiome interactions, and multi-organ pharmacokinetics. Identified key problems encompass the integration of immunological and mechanical modules (now found in less than 10% of systems), attaining high-throughput (with over 96 devices still being rare), and incorporating real-time multichannel sensors (used in approximately 12% of platforms). In 2025 the FDA issued guidance formally permitting organs-on‐chip and organoid data to substitute for animal data in drug approval submissions. The NIH has launched the ORIVA (Office of Research Innovation, Validation, and Application) to validate human‐centric MPS models [92]. These moves accelerate the translation of OoCs data into clinical decision‐making.

In parallel with regulatory developments, the commercialization and industrialization of OoC technology has progressed substantially. Several companies have transitioned OoC platforms from academic prototypes to commercially available products with standardized protocols and validated applications. Emulate, Inc. offers a portfolio of organ-chips, including liver, intestine, lung, and kidney models, that have been employed in FDA pilot programs for predictive toxicology assessment. MIMETAS has developed the OrganoPlate platform, a membrane-free microfluidic system in a 96-well format that enables gravity-driven perfusion and is compatible with existing laboratory automation infrastructure, making it one of the most scalable commercial OoC systems currently available. Furthermore, the enactment of the FDA Modernization Act 2.0 in 2022 formally removed the requirement for animal testing prior to human clinical trials, permitting the use of alternative methods including OoC systems, which has provided a significant regulatory impetus for industrial adoption [93]. These commercial platforms, coupled with regulatory acceptance, are collectively accelerating the integration of OoC data into pharmaceutical development pipelines, although challenges related to inter-platform standardization, cross-laboratory reproducibility, and the establishment of universally accepted qualification criteria remain to be resolved.

## Limitations, future perspectives and emerging technologies

A major focus in MPSs is the development of integrated multi-organ platforms. Linking multiple tissue chips through shared perfusion and microvascular networks enables simulation of systemic physiology and pharmacokinetics beyond single-organ models. Recent body-on-a-chip platforms have integrated various human tissues (e.g., gut–liver–heart–lung axes) within a singular device. For example, the integration of 18 organ modules through a microfluidic vascular network has been shown to provide quantitative investigations of inter-organ drug metabolism and signaling. Importantly, continuous efforts integrate blood-tissue interactions and endothelial vasculature to enhance authenticity [92]. In contrast, the short lifespan of the cells in the device remains a significant barrier to OoCs, and it is more severe when immortalized cell lines are not used. Cell lines can provide an almost infinite supply of identical cells suitable for high-throughput experiments, despite the common belief that they usually lack the capacity to replicate tissue-specific functions with great accuracy. This enhances reproducibility of outcomes, which would make it extremely beneficial for early drug development [18]. Concurrently, the integration of immunological and neurovascular components is progressing rapidly. Immunocompetent chips now incorporate circulating leukocytes and inflammatory signals to simulate responses to infection or immunotherapy, while hybrid organoid-chip systems are being developed to investigate organ-specific immunity [94]. Similarly, engineered neurovascular units and vascularized brain organoids are emerging, facilitating the co-culture of neurons with perfusable endothelium and microglia. These models aim to replicate the blood-brain barrier and neuroimmune interactions observed in vivo [95].

Despite these promising advances, several persistent limitations must be critically addressed before organoids and MPSs can achieve widespread translational implementation. Organoid immaturity remains to be a significant challenge: transcriptomic and functional analysis consistently show that most organoids, irrespective of tissue type, reach fetal-to-neonatal maturation states instead of adult phenotypes, even after extended culture periods, which restricts their predictive value for modeling adult-onset diseases and drug responses in mature tissues [24, 26, 36]. The absence of functional vasculature in conventional organoid cultures restricts oxygen and nutrient diffusion to approximately 200 μm from the organoid surface, resulting in hypoxic and necrotic cores in larger constructs that compromise cellular viability and architectural integrity. Although recent vascularization strategies, including co-culture with endothelial cells, microfluidic perfusion, and bioprinting of vascular channels, have shown partial success, achieving a fully perfusable, hierarchical vascular network within self-organizing organoids remains an unresolved challenge [7, 54].

Batch-to-batch variability presents a significant challenge, as the unpredictable nature of self-organization results in substantial variations in organoid size, morphology, cellular composition, and differentiation outcomes across production batches and among laboratories. This variability compromises the quantitative reproducibility necessary for standardized drug screening and regulatory acceptance. Current efforts to address it through defined synthetic matrices, guided differentiation protocols, and automated production systems have resulted in only slight advancements [35]. For microphysiological systems, the absorption of small hydrophobic drug molecules by PDMS, the most widely used fabrication material, can reduce effective drug concentrations by 20–80% depending on compound lipophilicity, thereby distorting dose-response relationships and undermining the pharmacological relevance of on-chip assays [51]. Alternative materials such as COC, glass, and thermoplastic polymers are being adopted to address this limitation, although each introduces distinct trade-offs in optical properties, gas permeability, fabrication complexity, and cost. In addition, the low throughput of existing OoC platforms remains a significant barrier to their incorporation into pharmaceutical screening workflows. Moreover, there is a notable absence of cross-platform standardization among various commercial OoC systems, which makes it challenging to compare and reproduce results across different laboratories and studies.

Another significant trend is the incorporation of on-chip sensors and computational analytics. Modern organ-on-chip systems increasingly integrate embedded biosensors (electrical, electrochemical, optical) enabling real-time, noninvasive assessment of tissue functionality. Integrated impedance electrodes or fluorescent metabolite sensors can continually monitor barrier integrity, oxygen consumption, or biomarker levels within the chip environment, producing more comprehensive data streams than endpoint assays [96]. Artificial intelligence and machine learning are being applied to analyze high-dimensional imaging and molecular datasets generated by organoids and MPS, optimize experimental design, and guide chip architecture development. Furthermore, machine learning methodologies are increasingly being applied to evaluate extensive phenotypic data from chips and organoids, optimize experimental design, and inform the development of novel chip architectures [11, 97, 98]. Conventional organ chips frequently experience low throughput; nevertheless, engineering advancements are producing high-density, automated platforms. Developers have implemented multi-well microfluidic arrays (e.g., 96- or 384-chamber chips) and multiplexed pumping systems to facilitate the simultaneous execution of experiments. Robotic liquid handling and compatible plate formats now enable the parallel culturing and assaying of hundreds of chip units, integrating with traditional high-throughput screening procedures [99].

## Conclusion

The evolution from 2D monolayer cultures to 3D organoids and advanced microfluidic MPSs represents a fundamental transformation in cell culture technology. As this review has critically assessed, each platform along this trajectory offers distinct advantages and limitations that determine its suitability for specific applications. The 2D monolayer cultures, despite their well-documented inability to recapitulate tissue architecture and in vivo microenvironments, retain irreplaceable utility for high-throughput primary screening, mechanistic pathway dissection, and standardized regulatory toxicology assays where simplicity, cost-effectiveness, and reproducibility are paramount. Organoids have advanced the field by capturing tissue-level self-organization, cellular diversity, and patient-specific disease phenotypes, yet their translational potential remains constrained by persistent immaturity, the absence of functional vasculature, batch-to-batch variability, and limited scalability. MPSs introduce dynamic perfusion, mechanical stimuli, and inter-organ communication that are essential for modelling systemic pharmacokinetics and biomechanical responses, although challenges related to PDMS drug absorption, low throughput, cross-platform standardization, and the high cost and complexity of device fabrication continue to restrict their widespread adoption. Despite ongoing challenges, MPS/OoCs are rapidly advancing, supported by regulatory attention and incorporation into translational workflows. These systems are poised to transform regenerative medicine and precision therapies by providing highly functional, human-relevant models that overcome many limitations inherent in conventional in vitro or animal-based models.

## Data Availability

No datasets were generated or analysed during the current study.

## Abbreviations

2D: Two-dimensional
3D: Three-dimensional
BMP: Bone morphogenetic protein
CF: Cystic fibrosis
CFTR: Cystic fibrosis transmembrane conductance regulator
COC: Cyclic olefin copolymer
ECM: Extracellular matrix
EGF: Epidermal growth factor
ESC: Embryonic stem cell
FDA: Food and Drug Administration
FGF: Fibroblast growth factor
IBD: Inflammatory bowel disease
iPSC: Induced pluripotent stem cell
MPSs: Microphysiological systems
PDMS: Polydimethylsiloxane
PDO: Patient-derived organoid
PSC: Pluripotent stem cell
Wnt: Wingless/integrated signaling pathway
